# Dysregulation of miRISC Regulatory Network Promotes Hepatocellular Carcinoma by Targeting PI3K/Akt Signaling Pathway

**DOI:** 10.3390/ijms231911300

**Published:** 2022-09-25

**Authors:** Maheshkumar Kannan, Sridharan Jayamohan, Rajesh Kannan Moorthy, Siva Chander Chabattula, Mathan Ganeshan, Antony Joseph Velanganni Arockiam

**Affiliations:** 1 Molecular Oncology Laboratory, Department of Biochemistry, School of Life Sciences, Bharathidasan University, Tiruchirappalli 620024, India; 2 Department of Biotechnology, Bhupat and Jyoti Mehta School of Biosciences, Indian Institute of Technology Madras, Chennai 600036, India; 3 Molecular Cancer Biology Laboratory, Department of Biomedical Science, Bharathidasan University, Tiruchirappalli 620024, India

**Keywords:** HCC, miR-221, astrocyte elevated gene-1, angiogenesis, cell proliferation, regulatory genes

## Abstract

Hepatocellular carcinoma (HCC) remains the third leading malignancy worldwide, causing high mortality in adults and children. The neuropathology-associated gene AEG-1 functions as a scaffold protein to correctly assemble the RNA-induced silencing complex (RISC) and optimize or increase its activity. The overexpression of oncogenic miRNAs periodically degrades the target tumor suppressor genes. Oncogenic miR-221 plays a seminal role in the carcinogenesis of HCC. Hence, the exact molecular and biological functions of the oncogene clusters miR-221/AEG-1 axis have not yet been examined widely in HCC. Here, we explored the expression of both miR-221 and AEG-1 and their target/associate genes by qRT-PCR and western blot. In addition, the role of the miR-221/AEG-1 axis was studied in the HCC by flow cytometry analysis. The expression level of the AEG-1 did not change in the miR-221 mimic, and miR-221-transfected HCC cells, on the other hand, decreased the miR-221 expression in AEG-1 siRNA-transfected HCC cells. The miR-221/AEG-1 axis silencing induces apoptosis and G2/M phase arrest and inhibits cellular proliferation and angiogenesis by upregulating p57, p53, RB, and PTEN and downregulating LSF, LC3A, Bcl-2, OPN, MMP9, PI3K, and Akt in HCC cells.

## 1. Introduction

Hepatocellular carcinoma (HCC) is the most common cancer and the third leading cancer-related death with a poor prediction [1]. HCC is the fastest rising cancers, following others, and has the highest incidence in developing countries, such as India [2]. Hepatitis B virus (HBV) and hepatitis C virus (HCV) are major risk factors in developing human HCC, with approximately 50–80% of cases from HBV and 10 to 25% from HCV infections, respectively [3]. Non-alcoholic fatty liver disease (NAFLD) has a role in developing HCC caused by the accumulation of fat exceeding 5% of liver weight in the absence of alcohol. NAFLD is a non-viral risk factor for developing HCC worldwide that majorly causes liver damage that leads to HCC from liver cirrhosis [4,5]. Surgery is considered the primary treatment for HCC, and recent studies have reported that chemotherapy is the most suitable treatment for HCC [6]. Nevertheless, the overall survival time of drug-treated patients is merely 2–5 years [7]. The molecular studies and targeted therapies for the regulatory protein networks of the apoptosis, cell cycle, and angiogenesis progression could be promising for treating HCC.

MicroRNAs (miRNAs) are a class of short-term endogenous non-coding RNAs, having 18–25 nucleotides in length. miRNAs act as oncogenes or tumor suppressors that dysregulate their target gene expression at the post-transcription level [8,9]. miRNAs are considered key mediators in various biological processes, including cell proliferation, apoptosis, and angiogenesis by dysregulation of specific target mRNAs [10,11] or the signaling pathways, such as PTEN/PI3K/Akt [12]. It is targeted by several miRNAs, particularly miR-221/222 [13], miR-543 [14], miR-146b [15], and miR-181a/b-1 [16] through binding in the 3’ Untranslated Region (UTR). Furthermore, miRNAs are involved in the NAFLD progression in various cell types and are considered a potential biomarkers [4]. Recent studies suggest that both tumor suppressors and oncogenic miRNAs correlate with several human diseases and play a critical role in cancer initiation and progression, especially the miR-221, which acts as an oncogene and induces carcinogenic activities in various human cancers, including HCC [17,18,19]. However, miR-221 specificity and sensitivity in HCC are not yet completely explored.

Astrocyte elevated gene -1 (AEG-1), also called Metadherin (*MTDH*) or protein Lysine Rich CECAM1 (LYRIC), has been reported as a human immunodeficiency virus (HIV)-1-inducible gene in human fetal astrocytes [20,21]. AEG-1 is overexpressed frequently and acts as an oncogene in several cancers, including HCC, but the clinical level studies of AEG-1 in the tumor initiation and progression in HCC are still unclear. AEG-1 plays a crucial role in cancer metastasis by regulating cell invasion and migration, apoptosis, angiogenesis, and chemo-resistance. The AEG-1 oncogene promotes the cells as aggressive cancers from the normal condition by dysregulating the corresponding proteins through the signaling pathways, such as PI3K/Akt [22,23]. During the RISC Complex formation, AEG-1 works as a scaffold protein and activates the miRNA as Onco-miR, which leads to the activation or degradation of specific regulatory genes [24].

Several studies have proven that the ectopic expression of miR-221 regulates the HCC by individual or cluster [25,26]. Meanwhile, several tumor suppressor miRNAs regulate AEG-1, and emerging evidence revealed that the lack of targeting miRNAs’ expression during carcinogenesis leads to the activation of AEG-1 [25,27]. However, numerous studies analyzed the specific miRNA/oncogene and their regulatory network, especially miR-221-targeted genes (PHF2 [25], C1QTNF1-AS1 [26]), and AEG-1-targeted miRNAs (miR-375 [27,28] and miR-195 [29]) in HCC. Hence, the regulation of both oncogene–onco-miR cluster such as AEG-1/miR-221 axis on the HCC regulatory network is still limited. In this study, we aimed to investigate the miR-221/AEG-1 axis regulations and molecular mechanisms for the potential targets in HCC.

## 2. Results

### 2.1. Clinical Significance of AEG-1 and miR-221 in Hepatocellular Carcinoma Patients. - MiR-221 and AEG-1 Were Highly Expressed in Hepatocellular Carcinoma Patients

**Figure 1:**
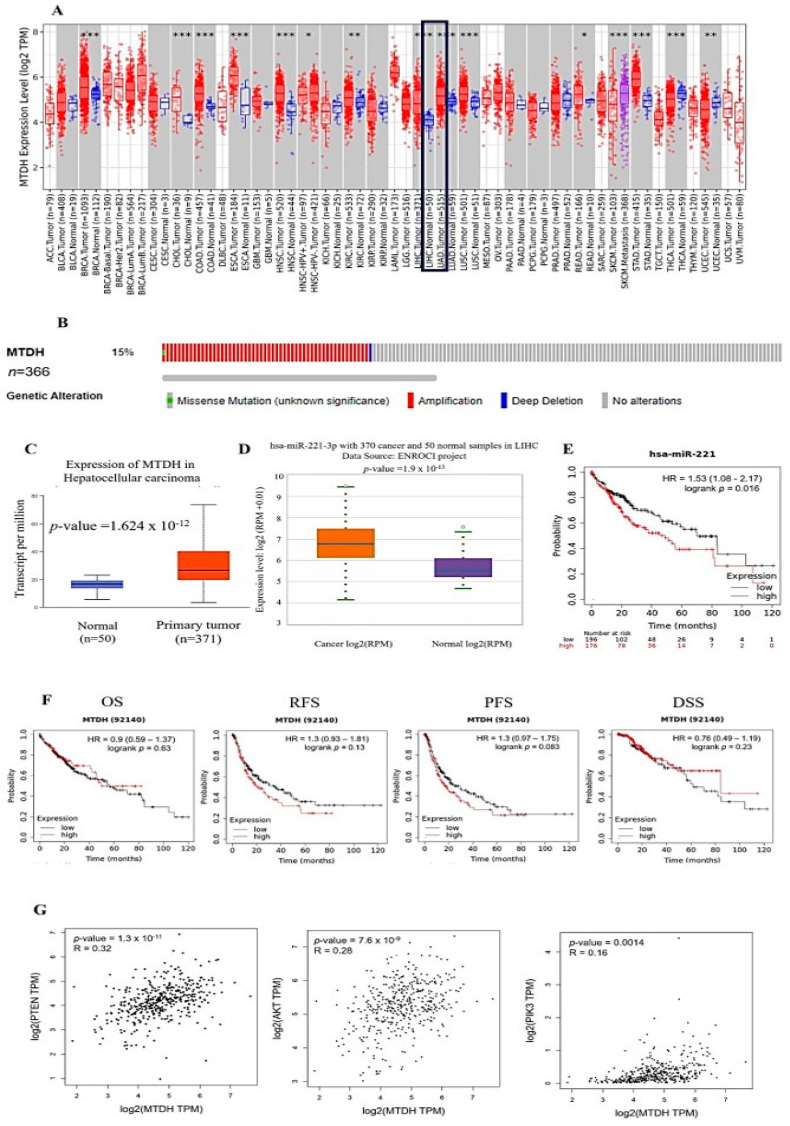
High expression of AEG-1 and miR-221 with poorer prognosis in liver cancer patients. (**A**). The mRNA expression of AEG-1 showed across all TCGA tumors, T (tumor—red) and N (normal—blue), Wilcoxon test used for statistical significance analysis (* *p*-value < 0.05; ** *p*-value < 0.01; *** *p*-value < 0.001) by using TIMER 2.0. (**B**) AEG-1 was amplified (red) in 15% of LIHC (liver hepatocellular carcinoma) (www.cbioportal.org) (accessed on 13 August 2022). (**C**) The AEG-1 mRNA expression was examined in normal (blue) (*n* = 50) and tumor (red) tissues (*n* = 371) from the cases of LIHC in the TCGA database using UALCAN (accessed on 13 August 2022). (**D**) miR-221 expression was examined in normal (blue) (*n* = 50) and tumor (orange) tissues (*n* = 370) from the cases of LIHC using the publicly available TCGA database using (http://starbase.sysu.edu.cn) (accessed on 13 August 2022) portal. (**E**) The Kaplan –Meier plot shows that a lower expression of miR-221 is associated with better overall survival of hepatocellular carcinoma patients. (**F**) Kaplan–Meier survival curves of liver cancer patients showed AEG-1 expression on overall survival (OS), relapse-free survival (RFS), progression-free survival (PFS), and disease-specific survival (DSS); the cutoff value of AEG-1 expression was set as its median value (accessed on 13 August 2022). (**G**) Pearson’s correlation between AEG-1 and PTEN, Akt, and PI3K expression in LIHC patients using the TCGA database using GEPIA 2 (Gene Expression Profiling Interactive Analysis) (accessed on 13 August 2022).

TIMER 2.0 (http://timer.cistrome.org/) (accessed on 13 August 2022) analysis showed that AEG-1 was upregulated in BRCA (breast invasive carcinoma), CHOL (cholangiocarcinoma), COAD (colon adenocarcinoma), ESCA (esophageal carcinoma), HNSC (head and neck cancer), KIRC (kidney renal clear cell carcinoma), LIHC (liver hepatocellular carcinoma), LUAD (lung adenocarcinoma), LUSC (lung squamous cell carcinoma), READ (rectum adenocarcinoma), STAD (stomach adenocarcinoma), THCA (thyroid carcinoma), and UCEC (uterine corpus endometrial carcinoma) (Figure 1A). AEG-1 was 15% amplified in LIHC (Figure 1B) (www.cbioportal.org) (accessed on 13 August 2022). Meanwhile, we investigated the expressions of AEG-1 and miR-221 in LIHC from TCGA data using the UALCAN and (http://starbase.sysu.edu.cn) (accessed on 13 August 2022) portal. Findings showed that AEG-1 and miR-221 were highly expressed in LIHC compared to normal tissues (Figure 1C,D). Next, we observed that low expressions of AEG-1 and miR-221 had high rates of OS (overall survival), RFS (relapse-free survival), PFS (progression-free survival), and DSS (disease-specific survival) in the Kaplan–Meier plotter (Figure 1E,F). The correlation of PTEN, Akt, PI3K, and AEG-1 mRNA expression was performed using GEPIA 2 plotter. The results showed that PTEN, Akt, and PI3K mRNA expression was significantly correlated with AEG-1 mRNA expression (Figure 1G). Altogether, these results suggest that the low expression of AEG-1 and miR-221 was associated with a better prognosis in hepatocellular carcinoma patients.

### 2.2. Ectopic Expression of miR-221/AEG-1 in HCC Cell Line Panel

**Figure 2:**
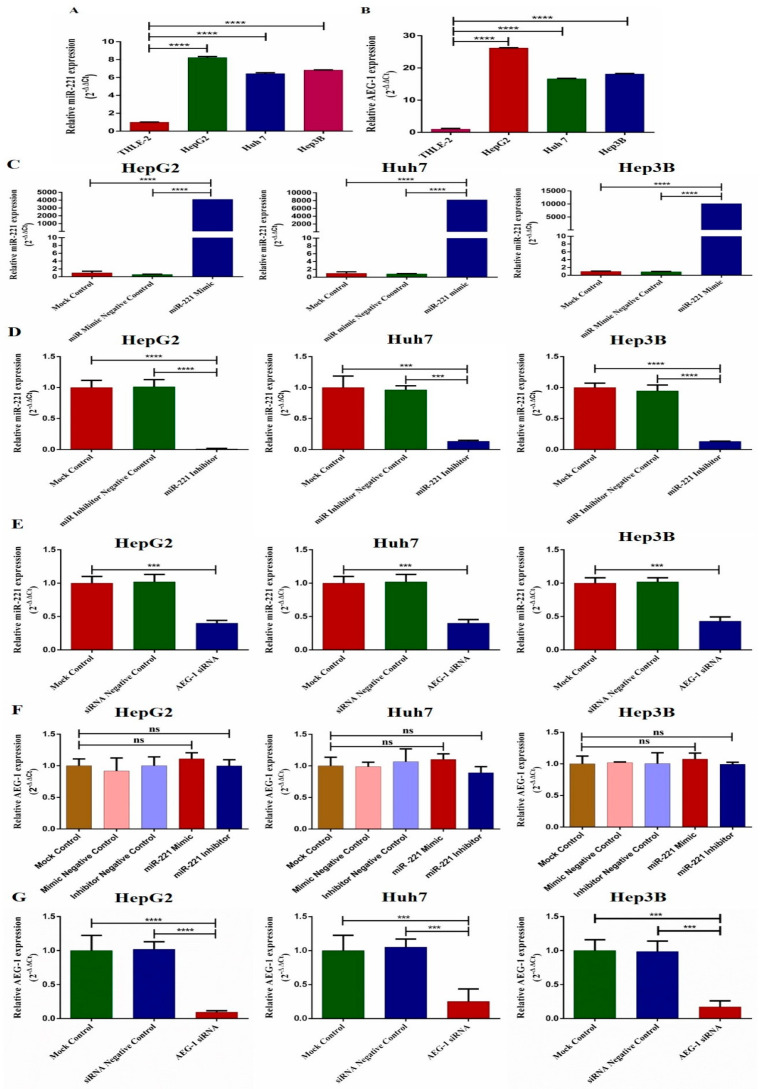
The relative miR-221 and AEG-1 mRNA levels in HCC cell lines. The miR-221 (**A**) and AEG-1 (**B**) relative mRNA levels analyzed in HCC cell line panels, the relative miR-221 expression in miR-221 mimic- (**C**), miR-221 inhibitor- (**D**), and AEG-1 siRNA- (**E**) transfected HCC cells. The relative mRNA levels of AEG-1 in miR-221 mimic/inhibitor- (**F**) and AEG-1 siRNA-transfected groups in HCC cells (**G**). The RNU6 and GAPDH were used as internal controls. Error bars presented as mean ± s.d and *p*-values represented as *** *p* < 0.001, **** *p* < 0.0001. ns represented as non-significance.

We investigated the miR-221 and AEG-1 expressions in normal human liver epithelial cells (THLE-2) and HCC cell line panels (HepG2, Huh7, and Hep3B) by qRT-PCR. The results revealed that the miR-221 and AEG-1 expressions were upregulated in all HCC cell lines compared to human liver epithelial cells (Figure 2A,B). Next, we analyzed the expression of miR-221 in the miR-221 mimic- (Figure 2C), miR-221 inhibitor- (Figure 2D), and AEG-1 siRNA- (Figure 2E) transfected groups. The miR-221 inhibitor- and AEG-1 siRNA-transfected groups showed decreased miR-221 expression, and the miR-221 mimic-transfected group showed ectopic expression of miR-221. Moreover, we examined the role of miR-221 mimic, miR-221 inhibitor, and AEG-1 siRNA on AEG-1. The results showed that the miR-221 mimic and miR-221 inhibitor did not alter the AEG-1 mRNA levels (Figure 2F) and AEG-1 siRNA silenced the AEG-1 mRNA levels in HCC cells (Figure 2G).

### 2.3. miR-221/AEG-1 Axis Regulates Apoptosis, Cell Cycle, Angiogenesis, and Autophagy Mechanism by the Activation of Regulatory Genes in HCC Cells In Vitro

**Figure 3:**
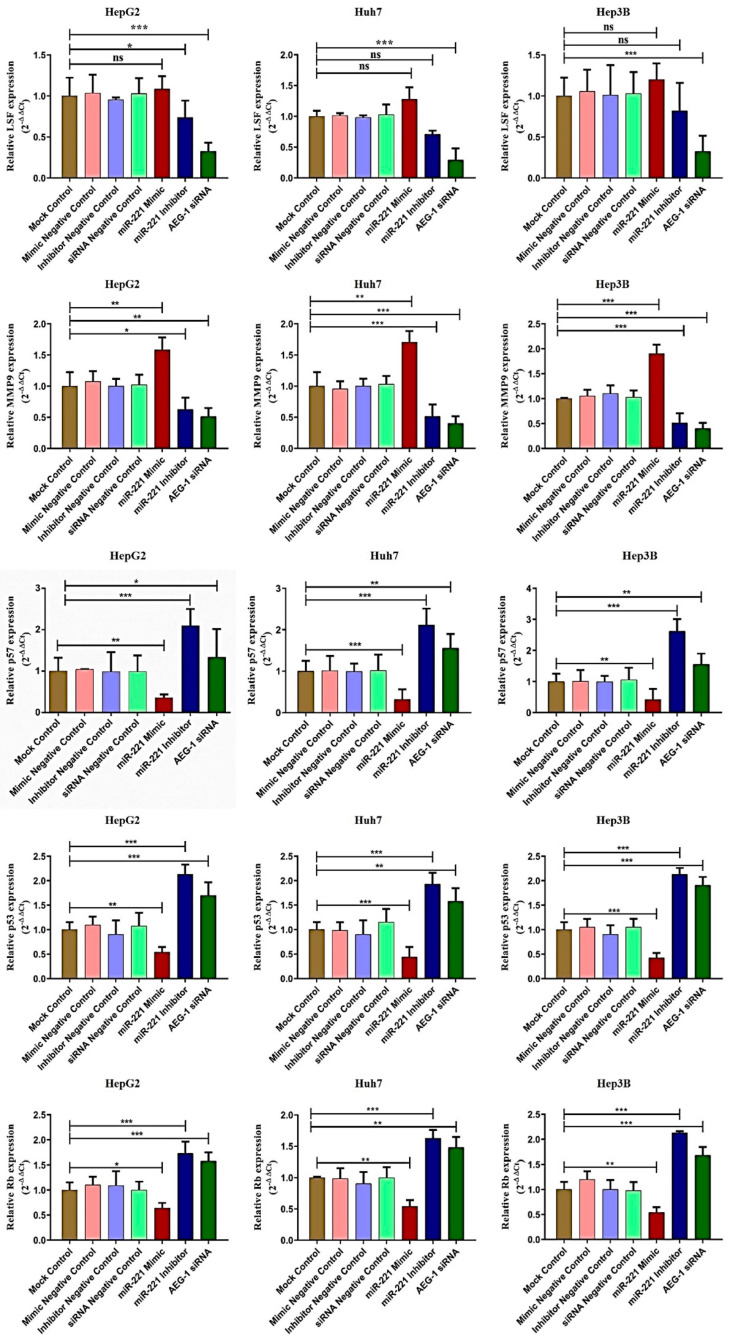
AEG-1 and miR-221 regulate angiogenesis and cell cycle regulatory mRNA expressions in the HCC cell lines. The regulatory mRNAs expressions which regulate angiogenesis (LSF and MMP9) and cell cycle (p57, p53, and RB) were analyzed in miR-221 mimic-, miR-221 inhibitor-, AEG-1 siRNA-, and their corresponding control-transfected HepG2, Huh7, and Hep3B cells by using qRT- PCR. The GAPDH was used as an internal control. Error bars presented as mean ± s.d and *p*-values represented as * *p* < 0.05, ** *p* < 0.01, and *** *p* < 0.001. ns represented as non-significance.

**Figure 4:**
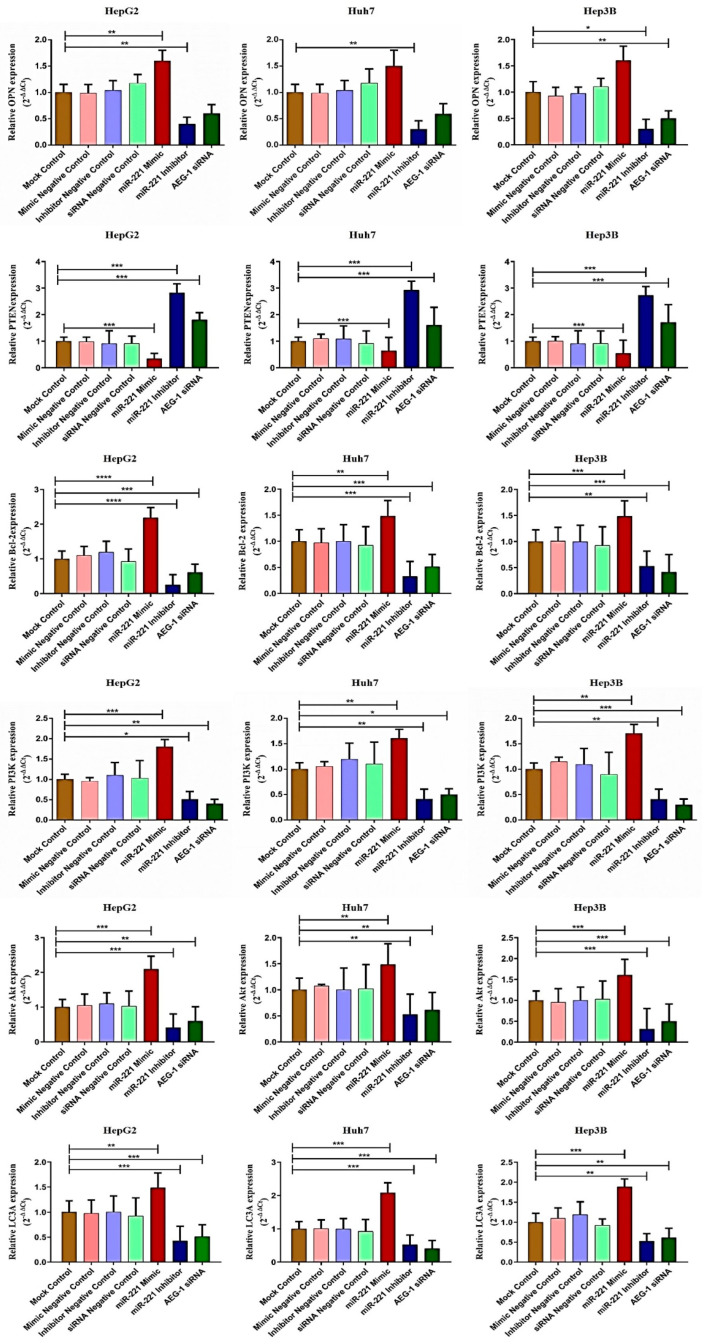
AEG-1/miR-221 regulates the apoptosis and autophagy regulatory mRNAs in the HCC cell lines. The apoptosis and autophagy regulatory mRNAs (OPN, Bcl-2, PTEN, LC3A, PI3K, and Akt) expressions were analyzed in miR-221 mimic-, miR-221 inhibitor, and AEG-1 siRNA-transfected HCC cells by using qRT-PCR, and GAPDH used as an internal control. Error bars presented as mean ± s.d. and *p*-value represented as * *p* < 0.05, ** *p* < 0.01, *** *p* < 0.001, **** *p* < 0.0001 compared with corresponding controls.

Next, we analyzed the AEG-1 and miR-221 roles in their regulatory network, which regulates cell cycle, apoptosis, angiogenesis, and autophagy in miR-221 mimic-, miR-221 inhibitor-, AEG-1 siRNA-, and their corresponding controls-transfected HCC panel by qRT-PCR. We observed the decreased expression of LSF, MMP9, OPN, Bcl2, LC3A, and PI3K/Akt and increased the PTEN, p57, p53, and RB expressions (Figure 3 and Figure 4) in the AEG-1 siRNA- and miR-221 inhibitor-transfected groups in HCC cells. The outcomes revealed that AEG-1 and miR-221 could play an essential role in the HCC regulatory networks.

### 2.4. Knockdown of miR-221 and AEG-1 Inhibits Invasion, Migration, and Cellular Proliferation In Vitro

**Figure 5:**
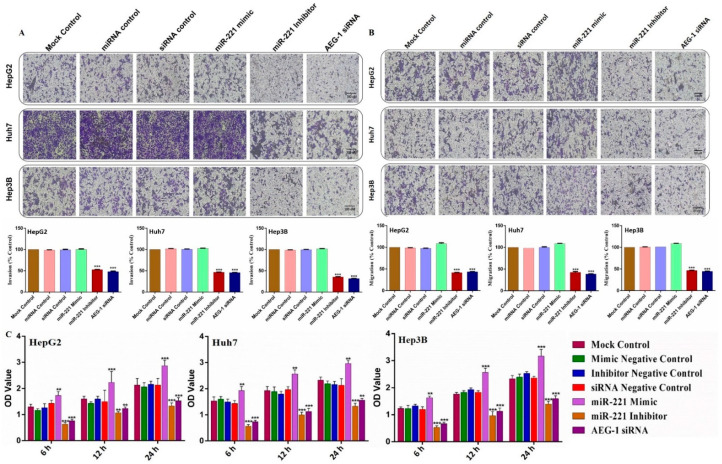
Silencing of AEG-1 and miR-221 inhibits HCC cell migration and proliferation in vitro. The effect of the miR-221/AEG-1 measured on HCC cell migration and cell proliferation in the miR-221 mimic-, miR-221 inhibitor-, AEG-1 siRNA-, and their control-transfected HepG2, Huh7, and Hep3B cells by invasion (**A**), migration (**B**), and MTT assay (**C**) in vitro. Images analyzed using Image J (NIH) (scale bar, 100 µm). Error bars presented as mean ± s.d and *p*-value represented as ** *p* < 0.01, *** *p* < 0.001 compared to the corresponding controls.

Next, we examined the effects of AEG-1 and miR-221 in the invasion, migration, and cellular proliferation activity by transwell assays, wound healing, and cell viability assays (MTT) in miR-221 mimic/inhibitor-, AEG-1 siRNA-, with their corresponding control- transfected HCC cells in in vitro. The results confirmed that the silencing of miR-221 and AEG-1 effectively inhibits HCC cell invasion (Figure 5A), migration (Figure 5B), and cellular proliferation (Figure 5C) (Supplementary Figure S1A–C) compared with their controls. These results confirmed that onco-miR-221 and AEG-1 oncogene are possible regulators of cell proliferation and migration in HCC cells.

### 2.5. Downregulation of miR-221 and AEG-1 Promotes Apoptosis and Cell Cycle Arrest in HCC Cells In Vitro

**Figure 6:**
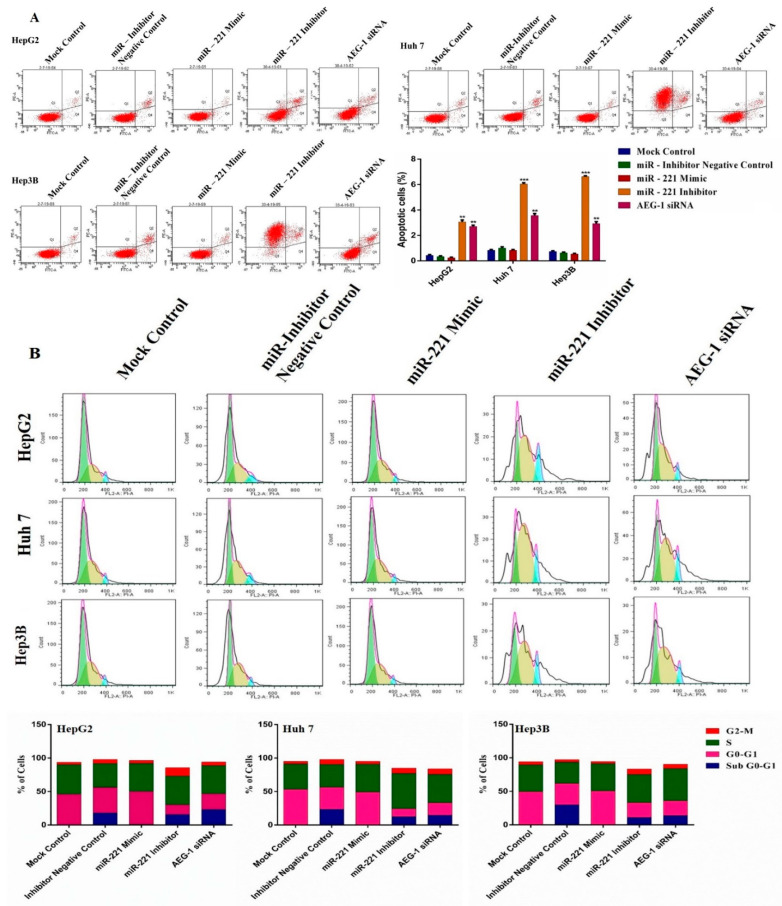
The effects of AEG-1/miR-221 on HCC cell cycle regulation and apoptosis. The effects of AEG-1/miR-221 on apoptosis (**A**) and cell cycle (**B**) analyzed in the miR-221 mimic-, and miR-221 inhibitor-, AEG-1 siRNA-, and their corresponding control-transfected HCC cells in vitro by flow cytometry analysis. Error bars presented as the mean ± s.d. and *p*-values represented as ** *p* < 0.01, *** *p* < 0.001 compared to the corresponding controls.

Furthermore, we performed flow cytometry analysis to confirm the miR-221 and AEG-1 regulation of cell cycle and apoptosis in HCC cells. HCC cells were treated with miR-221 mimic/inhibitor and AEG-1 siRNA and performed cell cycle analysis by PI staining and apoptosis assay by Alexa Fluor-conjugated Annexin V-FITC/PI dual-staining. The apoptosis (Figure 6A) and cell cycle (Figure 6B) results showed that the percentage of the apoptotic cells increased in G0-G1 and induced cell cycle arrest in sub-G1 and G2/M compared with miR-221 mimic and their corresponding control groups. These results proved that miR-221 and AEG-1 were involved and could control cell cycle regulation and apoptosis.

### 2.6. Downregulation of miR-221 and AEG-1 Inhibits Angiogenesis and Enhances Apoptosis and Cell Cycle Arrest by Modulating Regulatory Proteins In Vitro

**Figure 7:**
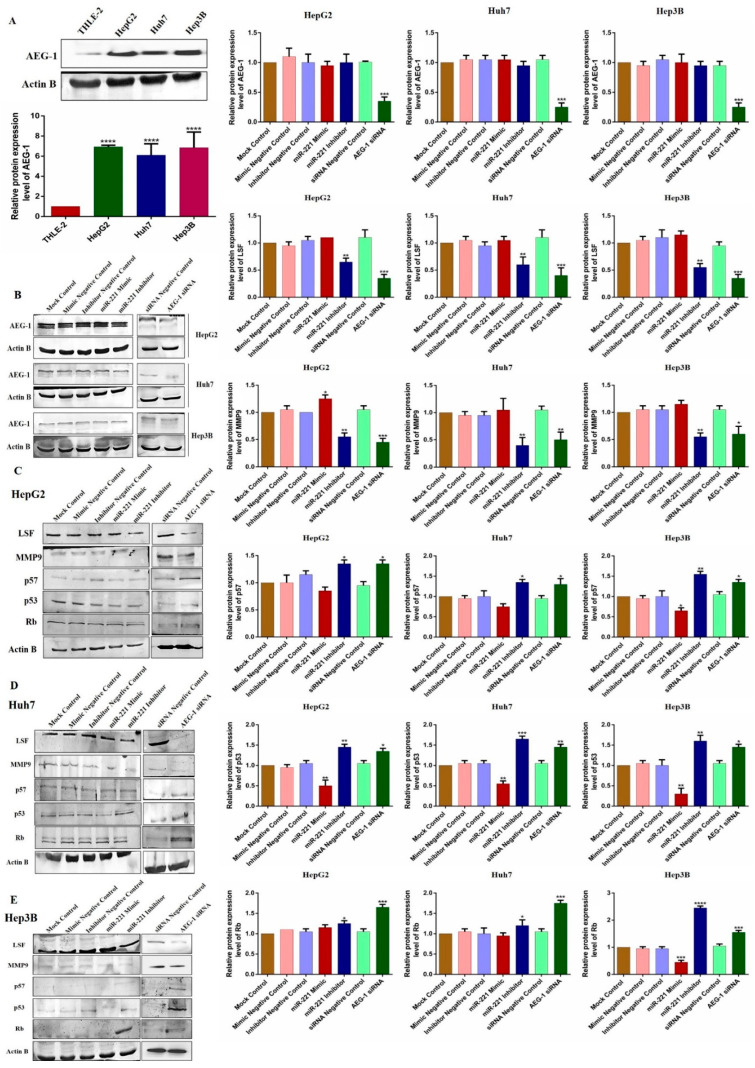
The effect of AEG-1/miR-221 was analyzed on cell cycle and angiogenesis regulatory proteins in HCC cell lines. The relative AEG-1 protein expression in THLE-2, HepG2, Huh7, and Hep3B cells (**A**) and miR-221 mimic-, miR-221 inhibitor-, and AEG-1 siRNA-transfected HCC cells (**B**). The regulatory protein expressions which regulate angiogenesis (LSF and MMP 9) and cell cycle (p57, p53, and RB) were analyzed in miR-221 mimic-, miR-221 inhibitor-, AEG-1 siRNA-, and their controls-transfected HepG2 (**C**), Huh 7 (**D**), and Hep3B (**E**) cells by western blot. *p*-Values presented as * *p* < 0.05, ** *p* < 0.01,*** *p* < 0.001, **** *p* < 0.0001 and error bars presented as the mean ± s.d. ns—(non-significant) compared to the controls.

We analyzed the AEG-1 protein expression HCC panel and confirmed that the relative protein expression of AEG-1 was overexpressed significantly in HepG2, Huh-7, and Hep3B compared to THLE-2 cells (Figure 7A). Following, the HCC cells were transfected with miR-221 mimic, miR-221 inhibitor, and AEG-1 siRNA, with corresponding controls to analyze the relative AEG-1 expression. The results showed that miR-221 mimic, miR-221 inhibitor, and their corresponding controls did not alter AEG-1 expression and significantly decreased in the AEG-1 siRNA-transfected group when compared to the control (Figure 7B).

**Figure 8:**
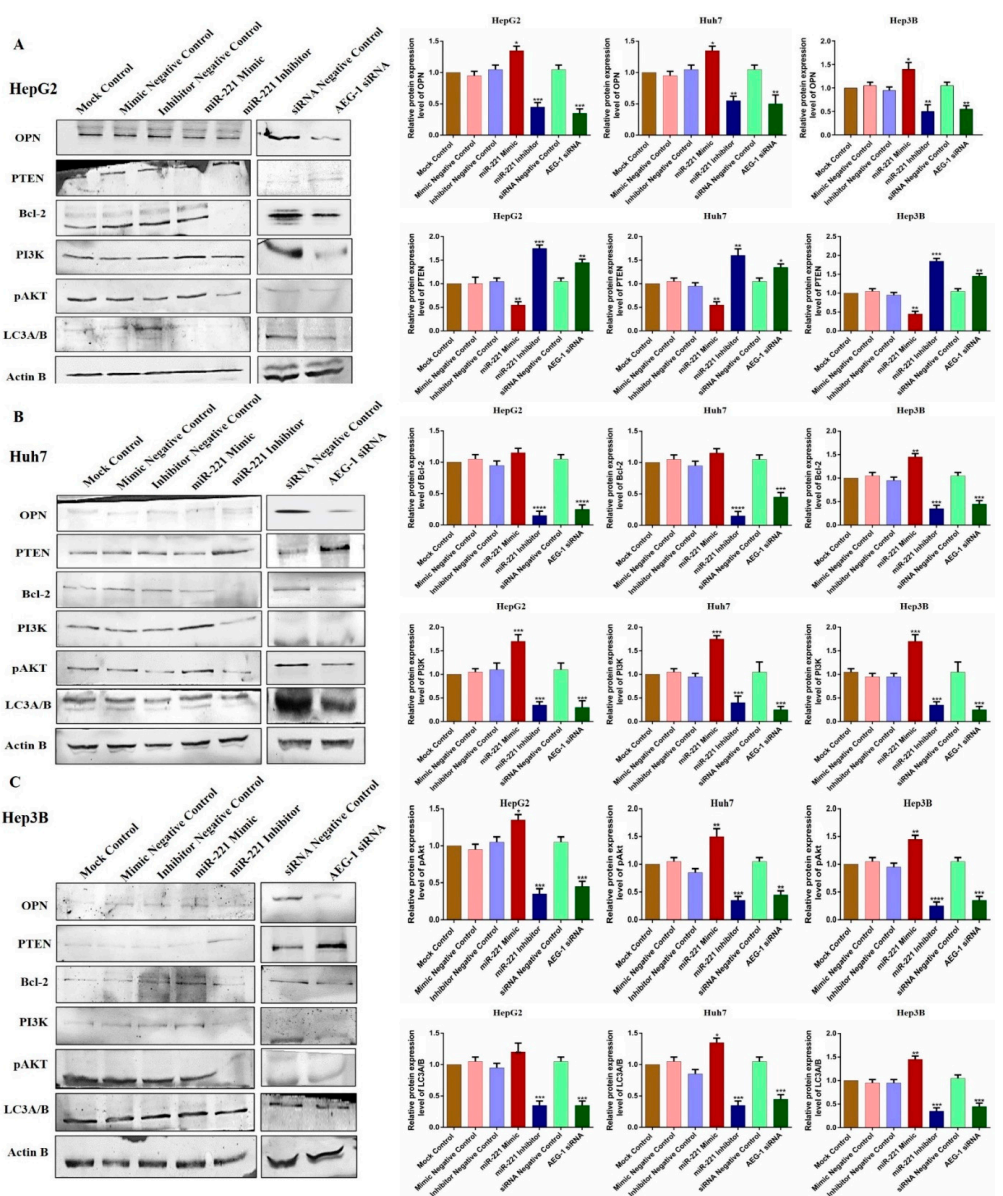
Ectopic expression of AEG-1/miR-221 dysregulates apoptosis and autophagy regulatory protein levels in HCC cell lines. The relative expressions of apoptosis (OPN and Bcl-2), autophagy (LC3A/B), PTEN, and PI3K/Akt proteins were analyzed by western blotting in the mock control, miR-221 mimic, miR-221 inhibitor, AEG-1 siRNA, and corresponding controls transfected HepG2 (**A**), Huh7 (**B**), and Hep3B (**C**) cells using β-actin as an internal control. Error bars are represented as mean ± s.d. and * *p* < 0.05, ** *p* < 0.01, *** *p* < 0.001, **** *p* < 0.0001 compared to the control group. ns: non-significance.

Furthermore, we investigated the impact of miR-221 and AEG-1 knockdown on regulatory proteins, which regulate the apoptosis, cell cycle, angiogenesis, and autophagy in HepG2 (Figure 7C), Huh7 (Figure 7D), and Hep3B (Figure 7E) cells. The results showed that the knockdown of miR-221 and AEG-1 significantly inhibited LSF and MMP9 and upregulated p57, p53, and RB protein levels when compared to the control. Moreover, we demonstrated the apoptosis and autophagy regulatory protein expression in miR-221 inhibitor- and AEG-1 siRNA-transfected groups of HepG2 (Figure 8A), Huh7 (Figure 8B), and Hep3B (Figure 8C) cells. The results indicated that the silencing of miR-221 and AEG-1 significantly increased PTEN protein levels and decreased OPN, Bcl-2, LC3A/B, PI3K, and p-Akt protein levels when compared to miR-221 mimic and control groups. Thus, we identified that miR-221 and AEG-1 are possible oncogenes in HCC.

## 3. Discussion

Recently, cancer has been the main challenge globally with limited treatment. Chemotherapy is considered a potential treatment that improves the survival of the patients. However, early cancer detection and prevention remain challenged because it is the only disease affecting the normal cell directly without any injury. In recent days, targeted therapies have been considered an advanced treatment for early detection and cancer metastasis [30]. HCC is one of the aberrant cancers in growing countries such as India. HBV, HCV, and NAFLD are the common risk factors for causing HCC [31], which stimulates cell proliferation by regulating several oncogenes or oncogenic miRNAs, especially AEG-1 [32] and miR-221 [33]. Astrocyte-elevated genes are one of the targets of the tumor-associated antigen (TAA), which also is a component of RISC that leads to regulating the miRNAs [20,21,34]. TAA is a class of tumor antigens that are overexpressed in cancer cells and observed in a lower expression in normal cells. TAA promotes tumorigenesis by regulating cell proliferation, chemoresistance, metastasis, and apoptosis in cancers like HCC [28]. These findings revealed that AEG-1 is frequently overexpressed in human cancers and associated with several hallmarks of HCC. We affirmed the previous findings that AEG-1 is overexpressed in HCC cells, which indicates that AEG-1 is a therapeutic hallmark for HCC.

The advanced evidence showed the miRNAs that contributes in several human pathological process, including cancers. The oncogenic or tumor suppressor miRNAs dysregulate the specific regulatory mRNAs, which are mainly involved in the malignant activities including metastasis, survival, proliferation, chemoresistance, and apoptosis in various cancers, including HCC. miR-221 is one of the onco-miRNAs that is significantly overexpressed in cancers and regulates cell proliferation and metastasis. Our findings revealed the aberrant expression of miR-221 and its regulation in HCC. These findings prove that miR-221 plays a crucial role in liver carcinogenesis.

Earlier studies confirmed that AEG-1 was targeted by some tumor suppressor miRNAs, especially miR-375 and miR-195. Also, it showed lower expression of these tumor suppressor miRNAs during carcinogenesis. The ectopic expression of miR-375 and miR-195 inhibited cell proliferation, angiogenesis, and cancer metastasis by targeting and inhibiting the AEG-1 expression in HCC [8,27,28,29] and cervical cancers [11]. However, the ectopic expression of onco-miR-221 enhanced cellular proliferation and metastasis in HCC through the dysregulation of their targeted oncogenes and tumor suppressor mRNAs [24,25,26]. Moreover, miR-221 enhanced the angiogenesis activity in HCC by upregulation of SND1 oncogene [35]. These findings confirmed that AEG-1 and miR-221 play major oncogenic roles and regulate human carcinogenesis, including HCC. The silencing of AEG-1 and miR-221 inhibits angiogenesis activity and cell proliferation in several cancers, including HCC. Therefore, we figured that the silencing of AEG-1 and miR-221 inhibits angiogenesis and cellular proliferation by AEG-1 siRNA and anti-miR-221 in HCC cells. The miRNAs play a vital role in cell cycle regulation and apoptosis and induce cell cycle regulation by targeting the regulatory mRNA during carcinogenesis. The ectopic expression of tumor suppressor miRNAs miR-875-5p and miR-375 enhanced G0 phase apoptosis by G1 Phase arrest. It confirmed that the upregulation of miR-875-5p [36] and miR-375 [9] induce apoptosis and cell cycle arrest through the silencing of AEG-1 in cervical cancer and HCC. Our results confirmed that the silencing of AEG-1 and miR-221 induces apoptosis in sub-G0-G1 and G2-M phase arrest and inhibits angiogenesis and cellular proliferation in HCC cells.

Previous studies revealed the onco-miR–oncogene cluster regulations and proved that the upregulation of onco-miR-26a and the CENTG1 oncogene enhances cell proliferation and metastasis in Glioma cancer cells [37]. However, the molecular mechanisms of the onco-miR/oncogene cluster and their correlations are still unclear and limited in human cancers, including HCC, especially the miR-221 and AEG-1 cluster. Therefore, we focused on the onco-miR–oncogene regulation and correlations in HCC, especially the miR-221 and AEG-1 axis. Also, we analyzed the miR-221 and AEG-1 expressions, correlations, and effects of this cluster on their regulatory network proteins, which involve apoptosis, cell proliferation, invasion, autophagy, and angiogenesis in HCC.

Several findings showed that oncogenes or onco-miRNAs target several oncogenes and tumor suppressor regulatory genes directly, or association through pathways. One of these oncogenes is the LSF/TFCP2 (transcription factor LSF) involved in the angiogenesis regulations and is overexpressed in human cancers, including HCC [38]. The LSF targets some downstream signal genes and different regulatory mRNAs, including OPN [39] and MMP9 [40]. Osteopontin (OPN) is a phospho-glycoprotein involved in tumor metastasis and cell death. The matrix metalloproteinase 9 (MMP9) is also responsible for cell growth and cancer metastasis. It helps tumor growth by enhancing angiogenesis regulation during carcinogenesis. The overexpression of LSF enhances the angiogenesis activity, cell invasion, and migration in HCC cells. In addition, it was previously identified that the transcription factor LSF is a direct downstream target of AEG-1, and the LSF mRNA levels significantly increased during the overexpression of AEG-1 in HCC cells [41].

Interestingly, miR-221 regulates OPN in osteoblasts and induces the OPN protein levels during carcinogenesis [42]. On the other hand, the AEG-1 targets MMP2/9 in thyroid cancer cells and enhances cell invasion and migration by inducing the MMP2/9 [43]. Moreover, the upregulation of miR-221/222 regulates and induces the MMP-9 protein expression in pancreatic cancers [44]. Our outcomes confirmed that the LSF, OPN, MMP-9 mRNA, and protein levels decreased while silencing the miR-221 and AEG-1 in HCC cells. The miRNAs or oncogenes regulate their downstream target proteins through direct targets or signaling pathways. The signaling pathways playing critical roles and promoting carcinogenesis include tumor initiation and progression. NF-κB is an essential transcription factor, and several pathological and physiological processes regulate this pathway in human cancers, including cancer development and metastasis. The NF-kB is an aberrant expression in tumors by the miRNAs or some oncogenes through different molecular mechanisms [45], including AEG-1 [46] and miR-221 [47].

miRNAs play a crucial role in cell proliferation that controls or promotes cell differentiation by dysregulating the cell cycle or apoptosis regulatory proteins such as PTEN, p27, and p57 in human cancers. The PTEN is a tumor suppressor protein that helps prevent cell proliferation and induces cell cycle arrest to promote apoptosis in normal conditions (48,49). The cyclin-dependent kinase inhibitor 1B (P27) and cyclin-dependent kinase inhibitor 1C (p57) work as inhibitors of the cyclin/cyclin-dependent kinase (CDK) complexes in the different phases of the cycle to prevent cell differentiation and induce the cell cycle arrest. The ectopic expression of miR-221 regulates PTEN, inhibiting its expression in lung cancer [48] and inhibiting the p57 and p27 protein levels in HCC. During knockdown of the miRNA-221, the p57 and p27 expression level increases, resulting in induced cell death in HCC cells [49]. On the other hand, some findings showed that the AEG-1 oncogene targets PTEN and p57 and regulates their protein level in HCC [24]. Our results also prove that the PTEN, p27, and p57 expressions significantly increase while silencing the AEG-1 and miR-221 in HCC cells.

The activation of the NF-kB signaling pathway alters several regulatory proteins, including PTEN. The overexpression of NF-kB inhibits the transcription level of PTEN by the activation of anti-apoptotic protein Bcl-2 and promotes cell proliferation [50]. NF-kB inhibits cell death and promotes cell proliferation in prostate cancer by the overexpression of Bcl-2 [51]. Some previous studies confirmed that AEG-1 regulates Bcl-2 and significantly downregulates the Bcl-2 protein levels while silencing the AEG-1 in HCC [52]. Moreover, the miR-221 regulates Bcl-2 and BAX proteins, enhances the BAX protein level, and inhibits Bcl-2 while silencing the miR-221 in bladder cancer [53]. The PI3K/Akt is another essential signaling pathway that involves and regulates cancer activation and metastasis. The upregulation of PI3K/Akt induces MDM2-mediated p53 degradation by silencing PTEN protein levels in gastric cancer [54].

In cervical cancer, retinoblastoma (RB1) is a tumor suppressor protein regulated by PTEN. PTEN inhibits the PI-3 Kinase-mediated RB phosphorylation in cervical cancer by deregulating phosphatidylinositol 3,4,5 triphosphate Phosphorylation [55]. Moreover, RB1 regulates NF-kB and inhibits NF-kB transcriptional activity in prostate cancer [56]. Autophagy is a mechanism considered essential for cancer cell survival and cell death. Most of the signaling pathways and regulatory genes are involved in the autophagy mechanism and regulate their function, including Bcl-2 [57], p53 [58], PTEN, PI3K/Akt [59], and NF-kB [60] in human diseases, including cancer. In this way, onco-miR-221 and AEG-1 clusters may regulate the apoptosis, cell cycle, angiogenesis, and autophagy regulatory proteins PI3K, Akt, p53, p57, Bcl-2, RB1, OPN, MMP9, and LSF in HCC. We obtained similar results in AEG-1 siRNA- and miR-221 inhibitor-transfected HCC cells.

Based on the earlier studies, both AEG-1 and miR-221 regulate several cancers, including HCC, by direct targeting or through signaling pathways. Hence, the interaction between the AEG-1 and miR-221 in HCC is not yet known. We identified a part of this, such as AEG-1 and miR-221 regulating cooperatively in HCC tumorigenesis.

## 4. Materials and Methods

### 4.1. Cell Lines, Cell Culture

Human HCC cell lines (HepG2, Huh7, and Hep3B) were obtained from the National Centre for Cell Science (NCCS), Pune, India, and cultured in DMEM with 10% FBS (fetal bovine serum) and 1% PS (penicillin streptomycin) (Invitrogen, Carlsbad, CA, USA). The normal liver epithelial cell line THLE-2 (ATCC-CRL-2706) was purchased from the American Type Culture Collection (ATCC, Manassas, VA, USA) and cultured in Bronchial Epithelial Cell Growth Medium (BMEM) with 0.08% phosphoethanolamine (Sigma Aldrich St. Louis, MO, USA), 10% FBS, and 0.06% epidermal growth factor (EGF), human recombinant (Corning, NY, USA). The cells were cultured in a 37 °C humidified chamber with 5% CO_2_.

### 4.2. miRNA and siRNA Transfection

HCC cells were cultured in a 6-well plate (1 × 10^6^) and transfected with miR-221 mimic, miR-221 inhibitor, and their control miRNAs (5 µM), small interfering RNA (siRNA) negative control, and AEG-1 siRNA (sense: 5′-GACACUGGAGAUGCUAAUAUU-3′, antisense: 5′-UAUUAGCAUCUCCAGUGUCUU-3′) (2 nM) as per the manufacture protocols using Lipofectamine 2000 and RNAi MAX reagents (Invitrogen, USA) in Opti-MEM medium (Thermo Fisher, Waltham, MA, USA). The mock control group was treated with transfection reagent alone in Opti-MEM (Without miRNAs and siRNAs). The transfection complex was prepared, and the cells were added directly to the transfection complex and incubated at 37 °C for 48 h. The transfection complex was removed in 6–8 h incubation and replaced with the fresh culture medium. The cells were collected and analyzed for the effects of miRNA and siRNA on the treated groups after 48 h transfection by quantitative real-time PCR (qRT-PCR) (Life Technologies, Burlington, Ontario, Canada).

### 4.3. Quantitative Real-Time PCR

Total RNA was isolated from the RNAi-transfected HCC cells using TRIZOL (Invitrogen, CA, USA). The 2 μg of total RNA was collected and randomly primed to synthesize the complementary DNA (cDNA) using the SuperScript First Strand cDNA Synthesis kit (K1622) (Thermo Fisher Scientific, Waltham, MA, USA) as per the manufacturer’s protocol. Following, we performed the qRT-PCR using Step One plus qRT-PCR system (Life Technologies, Burlington, ON, Canada) with SYBR™ Green Master Mix—Real-Time PCR Master Mix (Applied Biosystems, Waltham, MA, USA) to analyze the miR-221, AEG-1, LSF, MMP9, p57, p53, RB, OPN, PTEN, Bcl-2, PI3K, Akt, and LC3A mRNA expressions. The primers were purchased from Eurofins Genomics (Louisville, KY, USA) and listed in Supplementary Table S1. The U6 snRNA and GAPDH were internal controls for miR-221 and AEG-1. The data was collected, and the specificity of the primers was verified by melt curve analysis. The fold changes of miR-221 and mRNAs were calculated using the 2^−ΔΔCt^ method.

### 4.4. Transwell Migration and Invasion Assay

The transwell chamber (Corning) with the Matrigel-coated insert (2 mg/mL) was used for the invasion assay, and the Matrigel-free inserts were used for the migration assay. For invasion assay, cells were transfected with miR-inhibitor, miR-221 mimic, AEG-1 siRNA, and their corresponding controls for 48 h. Following the incubation, cells were collected and seeded (1 × 10^5^) in the upper chamber of the 8 μm transwell inserts with a serum-free DMEM. FBS (10%) containing DMEM was filled in the lower chamber and incubated for 48 h at 37 °C. After, the upper chamber was removed from the plate and fixed with methanol for 20 min at RT and stained with crystal violet (0.1 mg/mL). Following the staining, the non-invading cells were removed from the surface of the Matrigel, a cotton swab was used for removal, and the invading cells on the bottom of the upper chamber were imaged and counted using a light microscope with 20× magnification (Carl Zeiss, North York, ON, Canada). The same method was performed on the Matrigel-free membrane inserts.

### 4.5. Annexin V-FITC/PI Double Staining of Cell Apoptosis

The Annexin dual staining assay was performed to detect the apoptosis activity in HCC cells using Annexin V-FITC and propidium iodide (PI) (Invitrogen, Carlsbad, CA, USA). The cells were collected after 48 h incubation and washed twice with ice-cold phosphate buffer saline (PBS), and then the cells were resuspended in 100 µL of binding buffer containing Annexin V-FITC (5 µL) and PI (1 µL) (1 mg/mL) and kept in the dark (RT) for 15 min. Following the incubation, 400 µL of the binding buffer was added to each tube, and the cells were mixed well and analyzed within an hour using BD Accuri C6 Flow Cytometer (BD Biosciences, Franklin Lakes, NJ, USA). Flowjo^TM^ software (v.10.0.6) (Becton, Dickinson and Company, Ashland, OR, USA) was used for data analysis.

### 4.6. Propidium Iodide Staining of Cell Cycle Analysis

The flow cytometry analysis was performed to analyze the cell cycle using propidium iodide staining. After 48 h transfection, cells were collected and washed with ice-cold PBS twice and fixed with 70% ice-cold ethanol at −20 °C for 24 h. Following the incubation, cells were stained with 250 µL of propidium iodide (PI) containing RNAs staining solution (50 mg mL/1 mg) (MP Biomedicals, Santa Ana, CA, USA) and incubated at 4 °C in the dark for 30 min. Following the incubation, samples were mixed well and analyzed using FACS Calibur flow cytometry (BD FACS). FlowJo™ Software v.10.0.6 (Becton, Dickinson and Company, Ashland, OR, USA) was used for data analysis.

### 4.7. Wound-Healing Assay

The HCC cells were seeded in 12-well plates (1 × 10^5^) and transfected with RNAi. After the 48-h incubation, a wound was created on the surface of the monolayer using a 200 µL tip, and images were captured at different time intervals (0, 12, and 24 h) using Floid Cell Imaging Station (Life Technologies, Burlington, ON, Canada). The wound gap and width were measured by using ImageJ (v.1.46r) (National Institutes of Health, Bethesda, ML, USA).

### 4.8. MTT Assay

After 48 h of RNAi transfection, the HCC cells were collected and seeded in 96-well plates (1 × 10^4^). The cells treated with MTT ((3-(4,5-dimethylthiazol-2-yl)-2,5-diphenyltetrazolium bromide) (0.5 mg/mL) and incubated for 4 h at 37 °C. Following the incubation, the medium was removed and 150 uL of DMSO was added (Sigma-Aldrich, Burlington, MA, USA) to dissolve the crystals of MTT formazan and the plates were gently mixed for 5 min. The plate read at 570 nm using a microplate reader (Bio-Rad, Hercules, CA, USA) at different time intervals (0, 12, and 24 h).

### 4.9. Western Blotting Analysis

HCC cells were treated with the miR-221 inhibitor, miR-221 mimic, AEG-1 siRNA, and the corresponding controls. Following the 48 h, the cell lysate was collected and homogenized using RIPA (Radio Immunoprecipitation Assay) lysis buffer (Santa Cruz Biotechnology, Dallas, TX, USA). Lowry’s method was performed to estimate the protein. The 50 µg of total lysate was mixed with a 4 × SDS loading buffer at a 1:3 ratio, and samples were heated at 95 °C for 9 min. The samples were loaded and separated on SDS-polyacrylamide gels (12%) (100 V, 2.30 h) and then transferred onto a 0.2 µm nitrocellulose membrane (150 V, 1.30 h) (Bio-Rad, Hercules, CA, USA) by using Towbin buffer. The membrane was incubated with 5% BSA or skimmed milk powder (5% skimmed milk powder in 100 mL TBST (20 mM Tris, 136 mM NaCl, 0.1% Tween 20, (pH 7.6)) at RT to block the non-specific antibody binding. After 1 h incubation, the membranes were incubated with primary antibodies against AEG-1 (ab124789), Late SV40 factor (LSF) (ab80445), MMP9 (ab76003), LC3A/B (ab128025), Osteopontin (OPN) (ab91655), PTEN (ab32199), p57, Kip2 (ab75974), p53 (ab1101), RB (ab24), β-actin (ab6276), Bcl-2 (sc-7382), PI3K (sc-1637), and p-Akt (sc-514032) overnight at 4 °C. The membranes were washed with TBST and TBS 3 times (5 min/each) and incubated with alkaline phosphatase (ALP)-conjugated anti-mouse (ab97020) or anti-rabbit (ab6722) secondary antibodies for 2 h at room temperature. The BCIP/NBT solution (Merk, Kenilworth, NJ, USA) was used to detect the bands, and the densitometry of the band was analyzed using ImageJ (v.1.46r) (National Institutes of Health, Bethesda, ML, USA).

### 4.10. Statistical Analysis

GraphPad Prism (v 7.0) (GraphPad Software, San Diego, CA, USA) was used for statistical analyses, and one-way ANOVA was used for comparison. *p* < 0.05 was considered statistically significant, and all the data were performed and presented in at least three separate experiments.

## 5. Conclusions

We investigated and proved that miR-221 and AEG-1 were overexpressed in HCC and enhanced the HCC tumorigenesis by the regulatory network and signaling pathways. Recent studies considered targeted gene silencing by oncogenic or tumor suppressor miRNAs for diagnosis and therapy. We showed both onco-miR-221/AEG-1 oncogene axis regulations and their role in HCC. Our results indicated that the miR-221 expression level decreased in AEG-1 siRNA-transfected group, but the miR-221 did not alter the AEG-1 expression level in HCC cells. Our results showed that the miR-221 interaction with AEG-1 through RISC activation of miR-221 may be associated with AEG-1 during RISC complex formation and regulation by optimizing or enhancing the miR-221 activity. This complex leads to the dysregulation of their target/associated genes in HCC tumorigenesis.

Moreover, the silencing of both miR-221 and AEG-1 significantly increased the p53, p57, RB, and PTEN expression levels and decreased BCL2, LSF, MMP9, LC3A, PI3K, and p-Akt mRNA and protein levels in HCC cells. These findings revealed that the AEG-1/miR-221 plays a crucial role during HCC tumorigenesis by direct targeting or through PTEN/PI3K/Akt signaling pathways. Thus, the AEG-1 and miR-221 describe a novel therapeutic strategy to treat HCC. However, the exact interaction between miR-221 and AEG-1 in RISC and their molecular mechanism remains unclear. Further studies on the relationship between miR-221 and AEG-1 are essential to confirm their regulation in vivo. Further, our future study will explore the miR-221 and AEG-1 effects on RISC in NAFLD or liver cirrhosis-associated HCC.

## Data Availability

Data not presented in full in the body of this paper can be obtained by writing to the corresponding author.

## Supplementary Materials

The following supporting information can be downloaded at: https://www.mdpi.com/article/10.3390/ijms231911300/s1.
