# Supplementary material for: Dysregulation of miRISC Regulatory Network Promotes Hepatocellular Carcinoma by Targeting PI3K/Akt Signaling Pathway
Source: Int J Mol Sci. 2022 Sep 25;23(19):11300. doi: 10.3390/ijms231911300 (PMC9569668; doi:10.3390/ijms231911300)
Supplement: Supplementary file 1 [file ijms-23-11300-s001.zip › ijms-1919826-supplementary.pdf]

Supplementary Materials

Supplementary Figure S1:

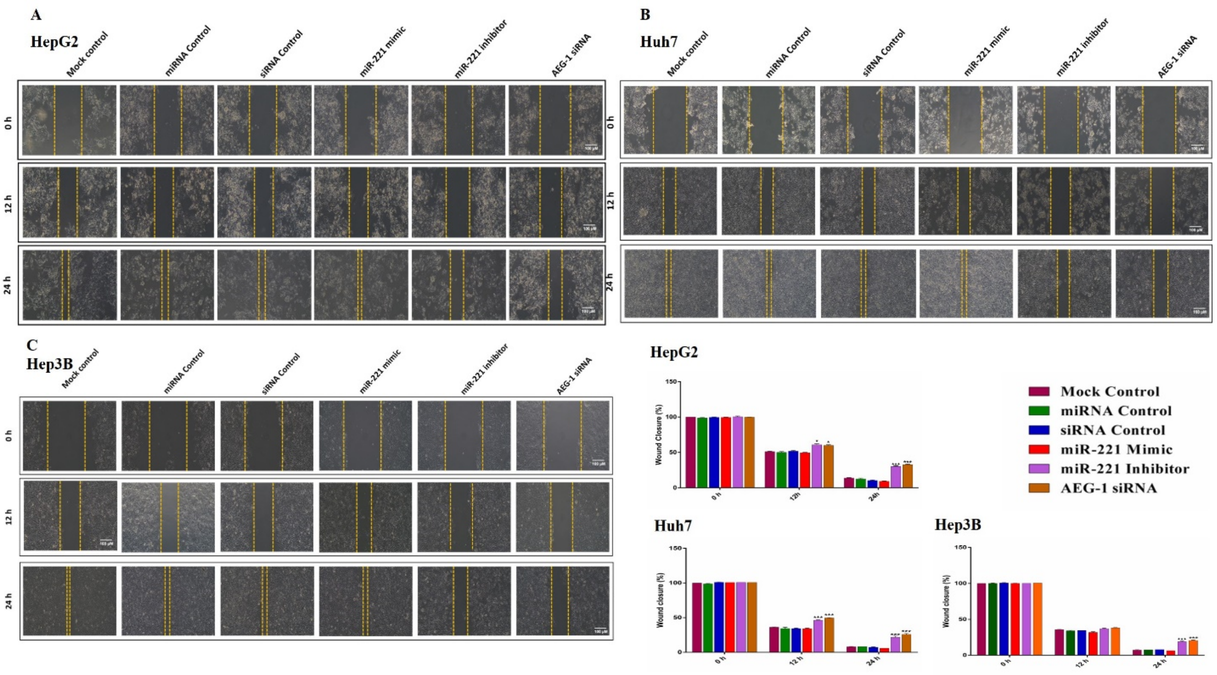

Supplementary Figure S1. Silencing of AEG-1 and miR-221 inhibits HCC cellular proliferation *in vitro*. The effect of the miR-221/AEG-1 measured on HCC cell proliferation in the miR-221 mimic, miR-221 inhibitor, AEG-1 siRNA, and their corresponding controls transfected HepG2, Huh7, and Hep3B cells by scratch assay (A, B, and C) *in vitro*. The wound gap was quantified in different time intervals (0, 12, and 24 h) using Image J (NIH) (scale bar, 100 μm). Error bars presented as mean ± s.d and P-value represented as **P<0.01, ***P< 0.001 compared to the corresponding controls.

Supplementary Table S1:

Primers List

miR-221 and U6 Reverse Transcription (RT) Primer

S.No	Gene	RT
**1**	miR-221	5’- GTCGTATCCAGTGCAGGGTCCGAGGTATTCGCACTGGATACGACGAAACC -3’
**2**	U6	5’-AACGCTTCACGAATTTGCGT-3’

qRT-PCR Primer

S. No	Gene	Forward Primer	Reverse Primer
1.	miR-221	5’- CGCAGCTACATTGTCTGCTGG-3’	5’- GTGCAGGGTCCGAGGT-3’
2	U6	5’-CTCGCTTCGGCAGCACA-3’	5’-AACGCTTCACGAATTTGCGT-3’
3	AEG-1	5’-AAATAGCCAGCCTATCAAGACTC-3’	5’-TTCAGACTTGGTCTGTGAAGGAG-3’
4.	LSF	5’-GGGTGCCATTCCGAGTACAA-3’	5’-GCCGAGTGTAAGTGCTCAGT-3’
5	MMP9	5’-CCTGGGCAGATTCCAAACCT-3’	5’-CAAAGGCGTCGTCAATCACC-3’
6	P57	5’-GTGAGCCAAAGCCCAAAGAG-3’	5’-TGCTACATGAACGGTCCCAG-3’
7	P53	5’-CCTCTCCCCAGCCAAAGAAG-3’	5’-TCTCGGAACATCTCGAAGCG-3’
8	RB1	5’-GTCCGGTTTTTCTCAGGGGA-3’	5’-GCGAGCTGTGGAGGAGC-3’
9	OPN	5’-TGACCCATCTCAGAAGCAGAA-3’	5’-CTTACTTGGAAGGGTCTGTGGG-3’
10	PTEN	5’-TGCGGTGACATCAAAGTAGAG-3’	5’-CTCTGGTCCTGGTATGAAGAATG-3’
11	Bcl-2	5’-GTGAACTGGGGGAGGATTGT-3’	5’-GGAGAAATCAAACAGAGGCC-3’
12	PI3K	5’- CCCCTCCATCAACTTCTTCA-3’	5’-CGGTTGCCTACTGGTTCAAT-3’
13	Akt	5’-GGGTTTCTCCCAGGAGGTTT-3’	5’-GTCCATGGTGTTCCTACCCA-3’
14	LC3A	5’-GACTCCATGGCTTCCGAGTT-3’	5’-GTCCACAGCTGCTTTTCCAC-3’
15	GAPDH	5’-ATGGGGAAGGTGAAGGTCG-3’	5’-GGGTCATTGATGGCAACAATATC-3’
